# Metastatic eccrine porocarcinoma: report of a case and review of the literature

**DOI:** 10.1186/1477-7819-9-32

**Published:** 2011-03-16

**Authors:** Ugo Marone, Corrado Caracò, Anna Maria Anniciello, Gianluca Di Monta, Maria Grazia Chiofalo, Maria Luisa Di Cecilia, Nicola Mozzillo

**Affiliations:** 1 Department of Surgery "Melanoma - Soft Tissues - Head & Neck - Skin Cancers", National Cancer Institute of Naples, Italy; 2 Department of Pathology, National Cancer Institute of Naples, Italy

## Abstract

Eccrine porocarcinoma (EPC) is a rare type of skin cancer arising from the intraepidermal portion of eccrine sweat glands or acrosyringium, representing 0.005-0.01% of all cutaneous tumors. About 20% of EPC will recur and about 20% will metastasize to regional lymph nodes. There is a mortality rate of 67% in patients with lymph node metastases. Although rare, the occurrence of distant metastases has been reported.

We report a case of patient with EPC of the left arm, with axillary nodal involvement and subsequent local relapse, treated by complete lymph node dissection and electrochemotherapy (ECT).

EPC is an unusual tumor to diagnose. Neither chemotherapy nor radiation therapy has been proven to be of clinical benefit in treating metastatic disease. Although in the current case the short follow-up period is a limitation, we consider in the management of EPC a therapeutic approach involving surgery and ECT, because of its aggressive potential for loregional metastatic spread.

## Background

Eccrine porocarcinoma (EPC) is a rare type of skin cancer arising from the intraepidermal portion of eccrine sweat glands or acrosyringium, being a primary tumor or, even more common, a malignant transformation of an eccrine poroma (EP), representing 0.005-0.01% of all cutaneous tumors [1]. In Europe, the incidence rate was < 0.28/100,000 [2]. It mainly occurs in the elderly, with equal incidence in both sexes. Approximatively less than 300 cases of EPC have been reported in medical literature since this disease was first described in 1963 [3-12]. About 20% of EPC will recur and about 20% will metastasize to regional lymph nodes [9]. There is a mortality rate of 67% in patients with lymph node metastases [13]. Although rare, the occurrence of distant metastases has been reported [5].

We report a case of patient with EPC of the left arm with axillary nodal involvement and subsequent local relapse. The etiology, diagnosis, management and prognosis of this disease are discussed, with a brief review of the literature.

## Case presentation

**Figure 1:**
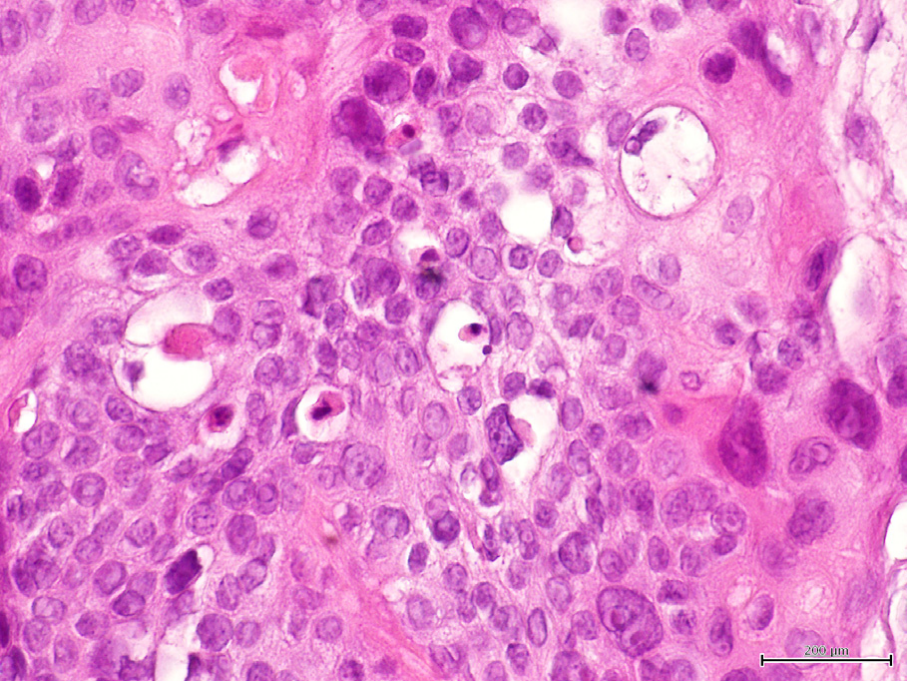
**Higher magnification revealing nests of epithelial tumor cells with a significant degree of cytologic atypia and mitotic activity (Hematoxylin and Eosin stain, ×60)**.

**Figure 2:**
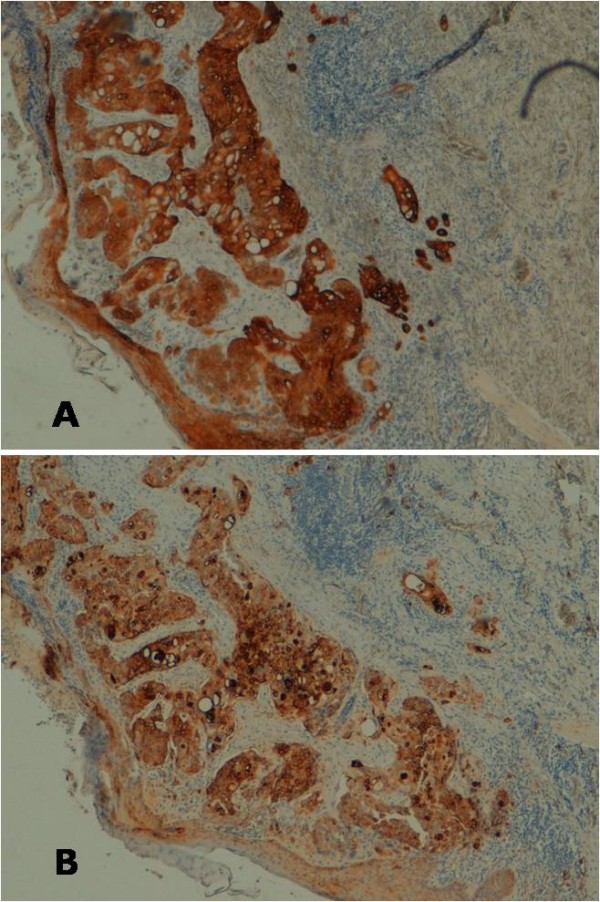
**Acrosyringeal differentiation confirmed by positive staining using antibodies to cytokeratins (CK, ×5) and  to epithelial membrane antigen (EMA, ×5) (A-B).** .

**Figure 3:**
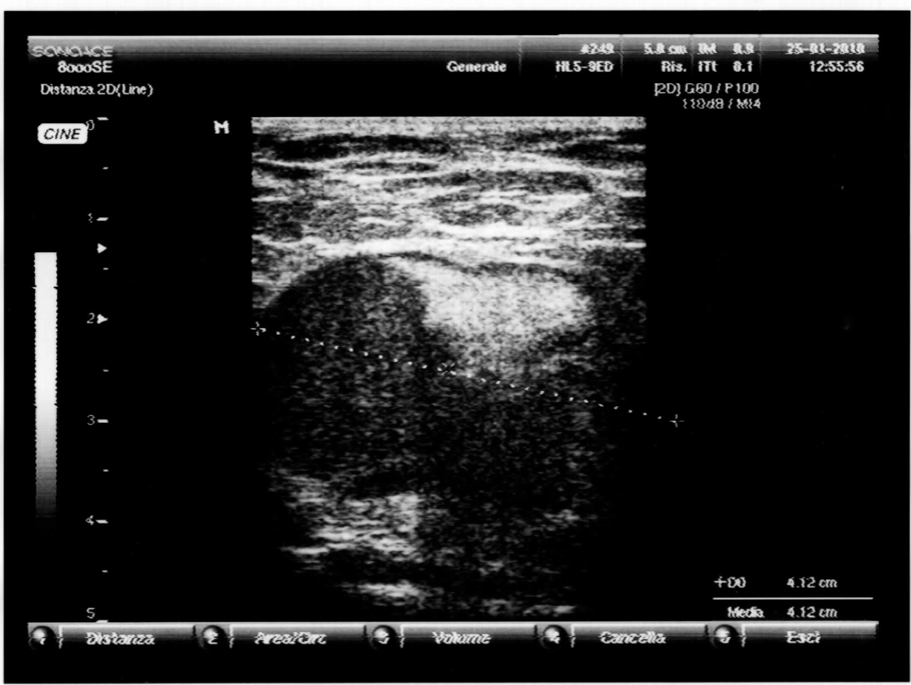
**US scan - Demonstration of axillary lymph node metastasis (4.1 × 2.5 cm)**.

**Figure 4:**
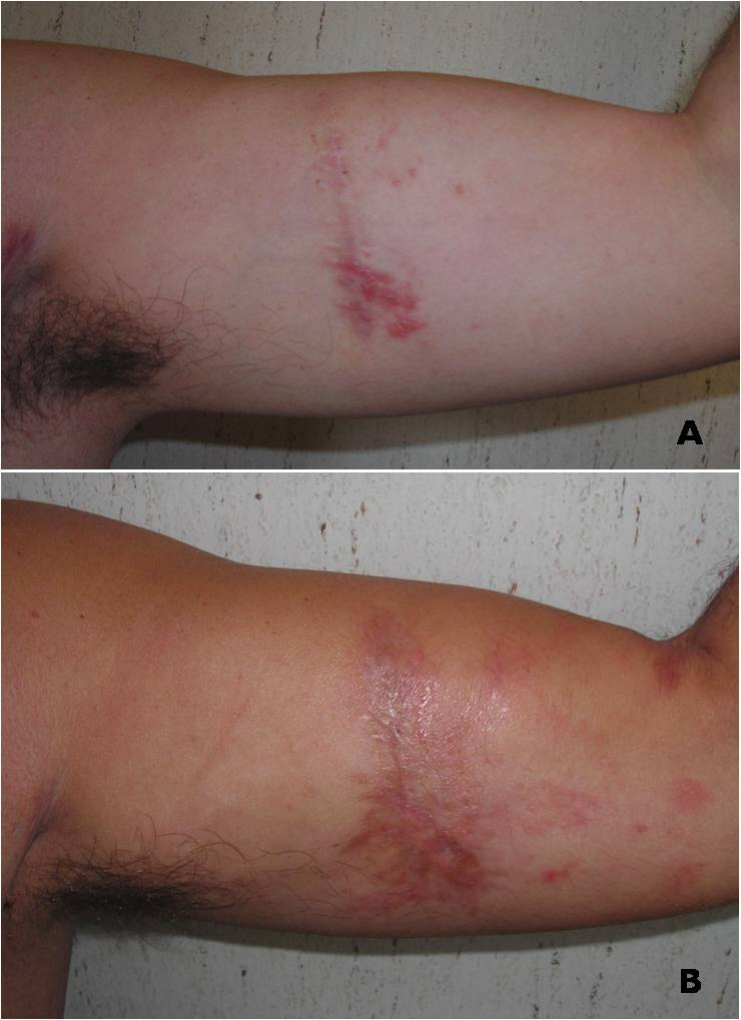
Local  relapse before ECT treatment and site of primary tumor after ECT treatment (A-B).

In February 2010 a 42-year old man presented with palpable left axillary lymphadenopathy. Ten months before this time point, he had been admitted to another institution for excision biopsy of an erythematous plaque less than 2 cm in size on the left arm with histological diagnosis of EPC. Then a further wide excision was undertaken to ensure adequate clearance and histological examination revealed no residual tumor. At our institution a histological reexamination of the primary lesion confirmed diagnosis of EPC (Figure 1) Immunohistochemical stains showed positive staining of the lesional cells with cytokeratins (CK) 7+/20-, epithelial membrane antigen (EMA) (Figure 2A-B). The tumor depth was 3.3 mm with mitotic activity of 14 mitoses per 10 high-power fields, and it showed lymphovascular invasion and Pagetoid intraepidermal extension. Preoperative staging included imaging with ultrasounds (US), revealing evidence of several involved nodes in the left axilla, the largest measuring 4.1 × 2.5 cm in diameter (Figure 3), whole body positron emission tomography (PET/CT), which showed uptake of the radiotracer in the left axilla (SUV 10) without evidence of other metastatic disease, and fine needle aspiration cytology (FNAC), which confirmed replacement by EPC. The patient underwent a complete axillary lymph node dissection, showing 6 metastatic nodes out of 28 examined. Three months after axillary dissection, diffuse erythematous-violaceous plaques, measuring less than 1 cm in diameter, presented around the scar of the primary tumor (Figure 4-A). Scraping cytology revealed features similar to the primary tumor and were treated by a session of electrochemotherapy (ECT). The procedure was performed under general anesthesia. Intravenous (iv) bleomycin at 15 U per m^2 ^of body surface (U/m^2^) was administered by a slow infusion in a time frame of 30 s to 1 min. Electric pulses were applied to the tumor lesions with a Cliniporator™device (IGEA srl, Carpi, Italy). Electrical parameters were the following: 8 pulses per run, of duration 100 μsec. and field strength of 1000 V/cm, were delivered 8 minutes after bleomycin injection, at the frequency of 5 kHz, by means of external electrodes N-20-HG. After a follow-up of five months, complete response of the local recurrence was observed on a clinically macroscopic basis (Figure 4-B), without any complications, well tolerated by the patient, which also presented at this time no signs of axillary relapse or systemic disease.

## Discussion

EPC is an infrequent cutaneous neoplasm arising from the cells of the acrosyringium with metastatic potential. This tumor may occur de novo or developing from a pre-existing lesion as degenerative progression, and it can manifest clinically as a solitary lesion with non characteristic macroscopic appearance, as an ulcerated nodule or as a plaque, polypoid, or verrucous lesion [14]. The most common location of EPC are the lower limbs, head and neck, trunk, vulva, breast, nail bed and upper extremities [15]. The histological diagnosis can be done on specific microscopic features. In the primary tumor, the malignant cells arise from the intraepidermal portion of the eccrine sweat glands and may be limited to the epidermis or may extend into the dermis. The tumor are asymmetric with cords and lobules of polygonal tumor cell, typically with a cribriform pattern. Nuclear atypia is evident, with frequent mitoses and necrosis. From the lymphatics, the tumor cells can invade the overlying epidermis because of the "epidermotropic" nature of the tumor cells (Pagetoid pattern) [16-18]. Immunohistochemical studies with positive staining using antibodies to various kinds of antigens (human CK, EMA, carcynoembrionic antigen, p53 protein and others) can be done to confirm acrosyringeal differentiation and to support the conclusive diagnosis [9]. The differential diagnosis of EPC is extensive and runs the spectrum of basal cell carcinoma to metastatic adenocarcinoma [15]. Histologic findings predictive of the aggressive clinical course were the evidence of lymphovascular invasion, which is associated with multiple regional cutaneous metastases, the existence of more than 14 mitoses per field and a tumoral depth of more than 7 mm [5]. In our case, the tumor depth was 3.3 mm with mitotic activity of 14 mitoses per 10 high-power fields, and it showed lymphovascular invasion and Pagetoid intraepidermal extension. Both regional and distant metastases are attributed to the tumor's ability to invade the dermal lymphatics. Solid organ metastases are observed in 10% of cases, lymph nodes metastases in 20% of cases, and local recurrence in 20% of cases [5-15]. However the prognosis of this carcinoma seems difficult to establish due to missed follow-up of cases described in the literature and tumor rarity.

The optimum surgical treatment for EPC is wide surgical excision of the primary tumor with broad tumor margins, given the propensity for local recurrences, with curative rates from 70% to 80% of cases [14-18]. Therapeutic lymphadenectomy should be performed in case of lymphadenopathy, while the role of sentinel lymph node biopsy (SLNB) for staging EPC remains unknown, and probably may be reserved in cases of histological aggressiveness or intralymphatic permeation by the primary tumor [16-20]. In our case, tumor cells were detected in the needle aspiration of the left axillary lymph node and an axillary lymphadenectomy was performed.

Electroporation, which can be used to introduce chemotherapeutic drugs directly into cancer cells (electrochemotherapy), has been shown in clinical trials to have a high response rate in treatment of patients with primary or metastatic skin cancers. The procedure is normally well tolerated by patients and can be repeated [21]. It should be considered as an excellent alternative to standard therapies in treatment of locoregional recurrent EPC.

Experiences with postoperative radiotherapy are also scarce. Its use is generally reserved for palliative care and tumor response is both partial and inconsistent [17-20].

No standard therapeutic protocols for metastatic EPC exist. However, a variety of chemotherapeutics have been used with varying degree of responsiveness. Gonzales-Lopez et al reported a case of a 71-year-old man developing multiple cutaneous and regional lymph node metastases 15 months after surgical excision of the primary tumor, treated with lymphadenectomy, radiotherapy, and oral isotretinoin, subsequently substituted by tegafur, with no evidence of distant metastases after a 5.6-year follow-up [16].

## Conclusions

EPC is an unusual tumor to diagnose. The treatment for the metastatic disease has not been standardized. Its early identification and complete excision gives the best chance of a cure. Neither chemotherapy nor radiation therapy has been proven to be of clinical benefit in treating metastatic disease. Although in the current case the short follow-up period is a limitation, we consider in the management of EPC a therapeutic approach involving surgery and ECT, because of its aggressive potential for loregional metastatic spread.

## Consent

Written informed consent was obtained from the patient for publication of this case report and accompanying images. A copy of the written consent is available for review by the Editor-in-Chief of this journal.

## Competing interests

The authors declare that they have no competing interests.

## Authors' contributions

UM conceived the study, carried out the literature search, and draft the manuscript; CC helped in management of the patient; AMA performed the histological analysis and provided histological sections as figures for manuscript; GDM helped in the preparation of the manuscript; MGC and MLDC carried out literature review and manuscript drafting; NM made critical revision and supervision. All authors read and approved the final manuscript.
